# RUNAT-BI: A Ruthenium(III) Complex as a Selective Anti-Tumor Drug Candidate against Highly Aggressive Cancer Cell Lines

**DOI:** 10.3390/cancers15010069

**Published:** 2022-12-22

**Authors:** Marta Albanell-Fernández, Sara S. Oltra, Marta Orts-Arroyo, Maider Ibarrola-Villava, Fany Carrasco, Elena Jiménez-Martí, Andrés Cervantes, Isabel Castro, José Martínez-Lillo, Gloria Ribas

**Affiliations:** 1 INCLIVA Biomedical Research Institute, Hospital Clínico Universitario Valencia, University of Valencia, 46010 Valencia, Spain; 2 Instituto de Ciencia Molecular (ICMol)/Departament de Química Inorgànica, University of Valencia, 46980 Valencia, Spain; 3 Center for Biomedical Network Research on Cancer (CIBERONC), 28029 Madrid, Spain; 4 Departament de Bioquímica i Biología Molecular, Facultat de Medicina, University of Valencia, 46010 Valencia, Spain

**Keywords:** antitumoral agent, ruthenium compound, 2,2’-biimidazole, cancer cell lines, apoptosis

## Abstract

**Simple Summary:**

To overcome some limitations of platinum-based chemotherapy agents, new active metallodrugs based on other transition metals are being researched. Runat-BI is a ruthenium-based compound which was synthesized and characterized at the University of Valencia. We investigated the in vitro effect of this compound on eight cell lines of different cancer types. Runat-BI reduced tumor growth and migration significantly in three cancer cell lines, showing selectivity for tumoral cells and little effect on non-tumoral ones. Its mechanism of action is still unknown but seems related to DNA synthesis as the cells with higher growth rates are the most affected by this chemotherapy agent. However, additional mechanism(s) likely play a role in its selectivity for several cancer cell lines. Moreover, Runat-BI slightly increases expression of the proapoptotic genes *BAX* and *CASPASE-3*. All these findings support its study as a potential anticancer therapy.

**Abstract:**

Ruthenium compounds have demonstrated promising activity in different cancer types, overcoming several limitations of platinum-based drugs, yet their global structure–activity is still under debate. We analyzed the activity of Runat-BI, a racemic Ru(III) compound, and of one of its isomers in eight tumor cell lines of breast, colon and gastric cancer as well as in a non-tumoral control. Runat-BI was prepared with 2,2’-biimidazole and dissolved in polyethylene glycol. We performed assays of time- and dose-dependent viability, migration, proliferation, and expression of pro- and antiapoptotic genes. Moreover, we studied the growth rate and cell doubling time to correlate it with the apoptotic effect of Runat-BI. As a racemic mixture, Runat-BI caused a significant reduction in the viability and migration of three cancer cell lines from colon, gastric and breast cancer, all of which displayed fast proliferation rates. This compound also demonstrated selectivity between tumor and non-tumor lines and increased proapoptotic gene expression. However, the isolated isomer did not show any effect. Racemic Runat-BI is a potential drug candidate for treatment of highly aggressive tumors. Further studies should be addressed at evaluating the role of the other isomer, for a more precise understanding of its antitumoral potential and mechanism of action.

## 1. Introduction

Cancer represents a major public health problem. With an estimated 19.3 million new cases worldwide in 2020, it remains among the main causes of death (10.0 million deaths) [1,2]. By percentage of new cases, the most frequent cancer types are female breast cancer with 11.7% (2.3 million), colon with 6% (1.1 million) and gastric with 5.6% (1.1 million) [2]. Cancer is defined as uncontrolled growth of abnormal cells anywhere in the body. It has traditionally been treated with surgery, chemotherapy, and radiation therapy; however, increasing studies in cancer research have extended new approaches such as immunotherapy, hormone therapy, gene therapy, and targeted therapy [3]. Nevertheless, treatment fails in certain tumors, predominantly due to metastasis, recurrence, heterogeneity, resistance to chemotherapy and radiotherapy and avoidance of immunological surveillance [4,5,6]. Current chemotherapy agents are greatly limited by their side effects and intrinsic or acquired drug resistance [7], prompting renewed research effort within the scientific community to obtain new highly effective agents for cancer treatment.

The most widely used drugs in conventional chemotherapy are platinum-based, among which cisplatin is one of the most commonly utilized to treat various solid cancers, either as a single agent or in combination. Cisplatin exerts anticancer activity via multiple mechanisms, mainly by generating DNA damage followed by activation of several signal transduction pathways which finally lead to apoptosis [8]. Despite the success of these compounds, drug resistance and several undesirable side effects have been reported, especially due to their lack of selectivity for cancerous over normal tissues. It is therefore of interest to seek out alternative therapeutic metal-based compounds that could overcome the limitations of these platinum-based drugs [9,10,11].

Ruthenium species have demonstrated promise in different cancer types, opening the door to the design of novel metal-based chemotherapeutic agents [9]. Those agents are most typically multitargeted, with specific activities against different cancers, a favorable toxicity profile and clearance properties, as they are eliminated from the kidneys, liver and bloodstream [7,12,13]. Ruthenium complexes such as RAPTA-C, NAMI-A and KP1019 have undergone preclinical or clinical trials for the treatment of different cancers [11,13]. KP1019 is a cytotoxic agent for the treatment of platinum-resistant colorectal cancers, whereas the non-cytotoxic NAMI-A is considered a very effective antimetastatic drug [14]. NAMI-A was the first ruthenium-based agent to enter phase I clinical trials, showing disease stabilization in patients with non-small-cell lung cancer. In a clinical phase-I/II study, however, the combination of NAMI-A with gemcitabine was less active than gemcitabine alone [12,15].

Global structure–activity relationships are still under debate for ruthenium metallodrugs as regards in vitro antiproliferative/cytotoxic activity and in vivo tumor-inhibiting properties as well as pharmacokinetics [12]. The mechanism of action of many of these complexes remains unclear. DNA was considered the main target of action [16]; however, RAPTA-C was found to bind exclusively on the histone proteins, unlike platinum metallodrugs [12,17]. KP1019 and KP1339, which are under clinical investigation, induced apoptosis predominantly by the intrinsic mitochondrial pathway [18]. The chaperone protein GRP78 (glucose regulatory protein 78) was its main target [19].

The increasing focus on and promising results shown by ruthenium-based antitumor agents led to our study and synthesis of new Ru(III)-based anticancer compounds. Among these, we selected Runat-BI to study antitumor activity, with the aim of analyzing its effect on viability, migration, cell doubling time (CDT) and expression of pro- and antiapoptotic genes in six breast cancer (BC) cell lines (HCC1937, HCC1500, HCC1806, MCF-7, MDA-MB-231, BT474), a colon cancer cell line (HCT116), and a gastric cancer cell line (AGS). The MCF10A cell line from a human mammary gland was used as the non-tumoral cell line control.

## 2. Materials and Methods

### 2.1. Reagents and Instruments

All starting chemicals and solvents used in the synthesis of Runat-BI were purchased from commercial sources and used without further purification. The elemental analyses (C, H, N) and X-ray microanalysis were performed through Central Support Service for Experimental Research (SCSIE) at the University of Valencia. The results of scanning electron microscopy (SEM-EDX) were obtained through a Hitachi S-4800 field emission scanning electron microscope with 20 kV of accelerating voltage and 9.0 mm of working distance. Electrospray Ionization Mass (ESI-MS) spectrum of Runat-BI was performed on a SCIEX TripleTOF 6600+ mass spectrometer by using a direct infusion electrospray ionization source (Figure S1). The infrared spectra (FT-IR) data for Runat-BI and 2,2’-biimidazole molecules were obtained through a Perkin Elmer Spectrum 65 FT-IR spectrometer in the 4000–500 range (cm^−1^) with 25 scans and a spectral resolution of 4 cm^−1^ (Figure S2). X-ray diffraction data collection was performed on a Bruker D8 Venture diffractometer with graphite-monochromated Mo-K_α_ radiation (λ = 0.71073 Å). Crystal parameters and refinement data, along with selected bond lengths and angles, for Runat-BI are summarized in Tables S1 and S2.

### 2.2. Cell Line Culture and Treatment 

Breast, gastric and colon cancer cell lines were obtained from the American Type Culture Collection (ATCC, Rockville, MD, USA). Cell lines were cultured in RPMI 1640 or DMEM medium supplemented with 1% L-glutamine and 10% fetal bovine serum (GIBCO, New York, NY, USA). The culture conditions were identical in all cell lines: 37 °C and 5% CO_2_. Cells were seeded for 24 h before treatment with Runat-BI or PEG (control) for MTT, wound healing and CDT (Table S3).

### 2.3. Cell Proliferation Assay

Cell proliferation was analyzed by colorimetric MTT [3-(4,5-dimethylthiazol-2-yl)-2,5-diphenyltetrazolium bromide] assay. We seeded and cultured 3000 cells in 96-well plates. Next, cells were treated with a specific drug dose and the MTT assay was performed 24, 48 and 72 h after treatment. DMSO was used as a dissolvent to solubilize the compound and convert the formazan into a colored solution. Absorbance of this colored solution was quantified by measuring through a spectrophotometer at 590 nm. Each experiment was performed in triplicate and repeated at least twice. Average values for triplicates were calculated. Absorbance observed at different drug concentrations was compared with the respective non-treated controls and cell viability was calculated. The IC_50_ was calculated using GraphPad Prism, with the R indicating the goodness of fit.

### 2.4. Scratch/Wound Healing Assay

Cells were seeded in 6-well plates at a density of 4 × 10^5^ cells/well and incubated overnight until they reached 70% confluence. A pipette tip was used to create a wound in the cell layer. Cells were then treated with Runat-BI (21 μM). Each experiment was performed in triplicate and repeated at least twice. Images were obtained at 0, 24, 48 and 72 h at the same position and closure area of migrating cells was measured using Image J software, and the percentage of wound closure was calculated and compared with time zero. The assay is particularly suitable for studying the effects of cell–matrix and cell–cell interactions on cell migration.

### 2.5. Cell Doubling Time (CDT)

We investigated the growth rate and CDT of the nine cell lines used in this study. On day 0, cells were plated in 6-well plates with an initial concentration of 1 × 10^5^ cells. Duplicate counts were made in a Neubauer chamber using Automated Cell Counter TC10TM (BioRad) every 24 h for 10 days after staining cells with trypan blue. Each cell line was cultured in duplicate. From the cell count data, the cell growth curve was constructed during the time studied and the CDT was calculated using the online tool http://www.doubling-time.com/compute.php (accessed on 20 March 2020).

### 2.6. RNA Extraction and Gene Expression by Real Time PCR (RT-qPCR)

Total RNA from all cell lines was isolated using High Pure RNA Isolation Kit from Roche following the manufacturer’s protocol. RNA was extracted from cellular pellet from around 1 × 10^6^ total cells. RNA concentration was measured using a NanoDrop ND 2000-UV-vis Spectrophotometer (Thermo Fisher Scientific Inc., Wilmington, DE, USA). cDNA synthesis was performed using the High-Capacity cDNA Reverse Transcription kit (Applied Biosystems TM by Life Technologies TM, Carlsbad, CA, USA). We used TaqMan^®^ Gene Expression Assays (Applied Biosystems^TM^ by Life Technologies^TM^, Carlsbad, CA, USA). Primers used in the assay were *BAX* and *CASPASA-3* (proapoptotic genes); *BCL-2* (antiapoptotic gene) and GAPDH as endogenous control, carried out by quantitative real time-PCR (qRT-PCR) in RNA extracted from cell lines. Normalization was performed with GAPDH. The data were managed using the QuantStudio™ Design & Analysis Software v1.4 (Applied Biosystems ™ by Life Technologies ™, Carlsbad, California, USA). Relative expression was calculated using the comparative Ct method and obtaining the fold-change value (ΔΔCt). 

### 2.7. Statistical Analysis

All statistical analyses were performed using R Bioconductor. Results were considered significant when *p*-value < 0.05. The IC_50_ analysis and graph representations were performed using GraphPad Prism.

## 3. Results

### 3.1. Preparation of Runat-BI

**Figure 1:**
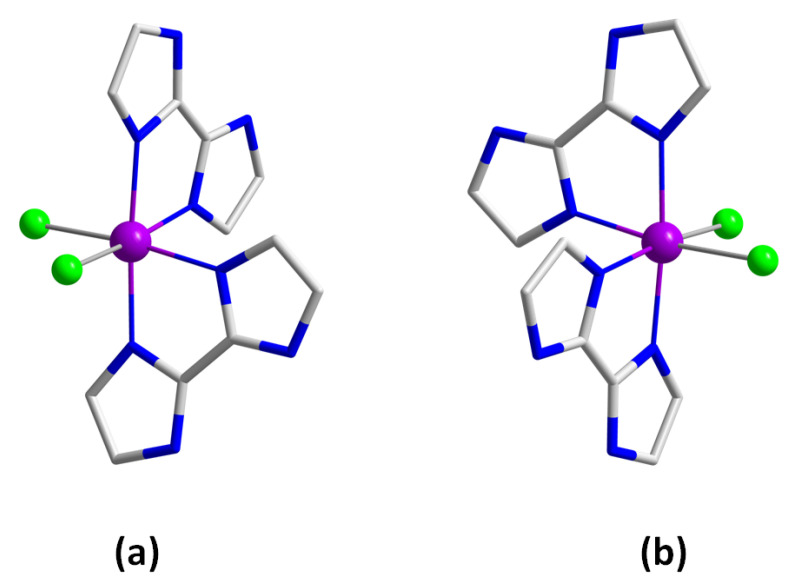
(**a**) Molecular structure of the mononuclear (inactive) isomer 1 of the {cis-[RuCl_2_(H_2_biim)_2_]Cl}_2_·4H_2_O racemic mixture in Runat-BI. (**b**) Molecular structure of the mononuclear isomer 2 of the {cis-[RuCl_2_(H_2_biim)_2_]Cl}_2_·4H_2_O racemic mixture in Runat-BI. Water molecules, hydrogen atoms and chloride counter anions were omitted for clarity.

RuCl_3_·H_2_O (6.6 mg, 0.03 mmol) and 2,2’-biimidazole (12.1 mg, 0.09 mmol) were reacted by means of a solvothermal synthesis in HCl (2.5 mL, 3.0 M) at 90 °C for 20.5 h, followed by a 20.5 h cooling process to room temperature. Dark blue crystals of Runat-BI were thus obtained and were suitable for X-ray diffraction data collection (Figure 1). Yield: ca. 30%. The same synthesis was performed in a concentrated solution and in a 1:1 ratio of the reactants, thus allowing us to isolate the isomer 1 (Figure 1). Anal. Calcd. for C_12_H_16_N_8_O_2_Cl_3_Ru: C, 28.2; H, 3.2; N, 21.9%. Found: C, 28.5; H, 3.3; N, 22.2%. SEM-EDX analysis gave a Ru:Cl molar ratio of 1:3 for Runat-BI. ESI-MS (*m*/*z*): 445.90 (4.8%), 444.93 (5.4%), 442.89 (16.4%), 441.82 (94.6%), 440.94 (41.9%), 439.97 (100%), 438.90 (62.0%), 437.93 (36.5%), 436.86 (30.6%), 435.98 (12.7%), 434.90 (3.1%), these *m*/*z* values support the stability of Runat-BI in solution (Figure S1). Infrared (IR) peaks (sample prepared as KBr pellets): 3278 (m), 3147 (m), 3129 (m), 3010 (m), 2923 (w), 2924 (m), 2765 (m), 1638 (s), 1526 (s), 1417 (m), 1394 (m), 1319 (w), 1252 (w), 1177 (m), 1129 (m), 1078 (m), 1008 (w), 922 (m), 870 (w), 811 (w), 754 (s) and 682 (m) cm^−1^ (Figure S2). 

### 3.2. Runat-BI Reduces Proliferation in Cancer Cell Lines 

**Figure 2:**
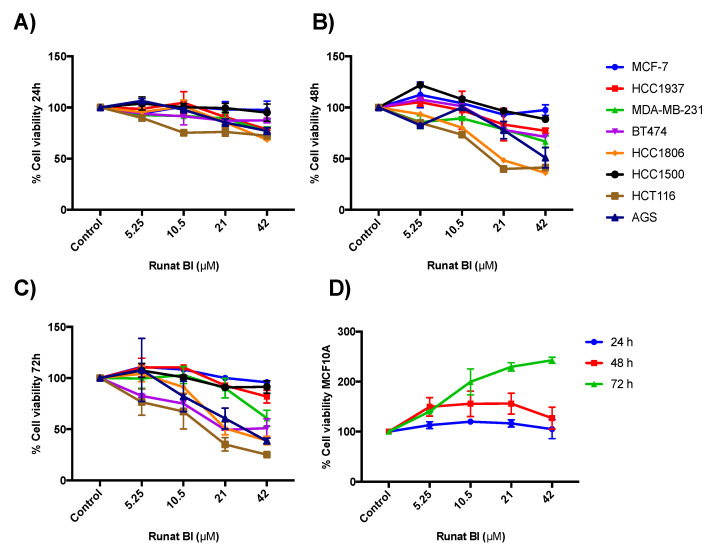
Viability percentages of the nine cell lines treated with Runat-BI at 24, 48 and 72 h. The breast cancer cell lines (MCF-7, HCC1937, MDA-MB-231, BT474, HCC1806, HCC1500), gastric cancer cell line (AGS) and colon cancer cell line (HCT116) were treated with Runat-BI at different concentrations (0 to 42 µM) for 24 h (**A**), 48 h (**B**), and 72 h (**C**). The mammary gland cell line MCF10A was used as a non-tumor cell line and was treated with Runat-BI at the same concentrations and time: 24 h (blue), 48 h (red) and 72 h (green) (**D**). Cell proliferation was determined with the MTT assay. Dots indicate the mean of three independent experiments.

Cytotoxicity of Runat-BI was analyzed in HCC1937, HCC1500, HCC1806, MCF-7, MDA-MB-231, BT474, HCT116, AGS and MCF10A cell lines by MTT assay. The nine cell lines used were subjected to increasing concentrations of Runat-BI (0, 5.25, 10.5, 21 and 42 µM in PEG) for 24, 48 and 72 h. As shown in Figure 2A–C, a dose- and time-dependent reduction in cell proliferation was observed for some cell lines. MTT assay showed significant viability reduction at 48 h for HCT116 and HCC1806 cell lines, reaching 41% and 36% viability, respectively, which presented the lowest IC_50_ (<25 µM) [Table S4]. At 72 h after treatment, we detected an around 50% reduction in BT474 and AGS cell line viability. The three lines (HCT116, HCC1806 and AGS) remained highly aggressive tumors with rapid growth and proliferation. In contrast, the MDA-MB-231 cell line displayed a moderate viability reduction (61%) after 72 h of treatment. Our results showed that Runat-BI compound is not active against HCC1937 (IC_50_ 94.23 µM), HCC1500 (IC_50_ 76.03 µM) or MCF-7 (IC_50_ > 200 µM), all belonging to BC cell lines; these were not significantly affected in terms of viability, which remained above 80% at 72 h. Table S4 displays the IC_50_ after 48 and 72 h of Runat-BI treatment. Nonetheless, viability of the non-tumor mammary gland cell line MCF10A was not reduced at any time or at any concentration (Figure 2D). The Runat-BI compound is not active against this cell line despite its rapid growth rate. These results support the specificity of the Ru(III) compound to affect tumor cells but not normal cell lines like MCF10A. 

### 3.3. Isomer 1 of Runat-BI Showed No Effect on Viability in the Cell Lines Studied

We selected the two cell lines AGS and MDA-MB-231, with different responses against Runat-BI compound, to test the isolated isomer 1 of Runat-BI. The concentrations tested for isomer 1 were slightly higher (0, 6.25, 12.5, 25 and 50 µM). Results showed no reduction in cell viability at any concentration or at any time (24, 48 and 72 h) [Figure S3]. In contrast to the viability reduction observed with Runat-BI, cells treated with isomer 1 showed increased viability at low concentrations of the compound after 48 and 72 h of treatment (Figure S3B,C). These findings showed no effect on isomer 1 from Runat-BI in the cell lines tested. These results open two potential hypotheses that could explain the Runat-BI effect in cancer cell lines: (1) The cytotoxic effect of the Runat-BI compound requires the presence of both isomers 1 and 2, or (2) the cytotoxic effect of Runat-BI is mainly attributed to isomer 2, which has not been isolated as yet.

### 3.4. Migration Reduction in Cell Lines Treated with Runat-BI Measured by Wound Healing Assay

**Figure 3:**
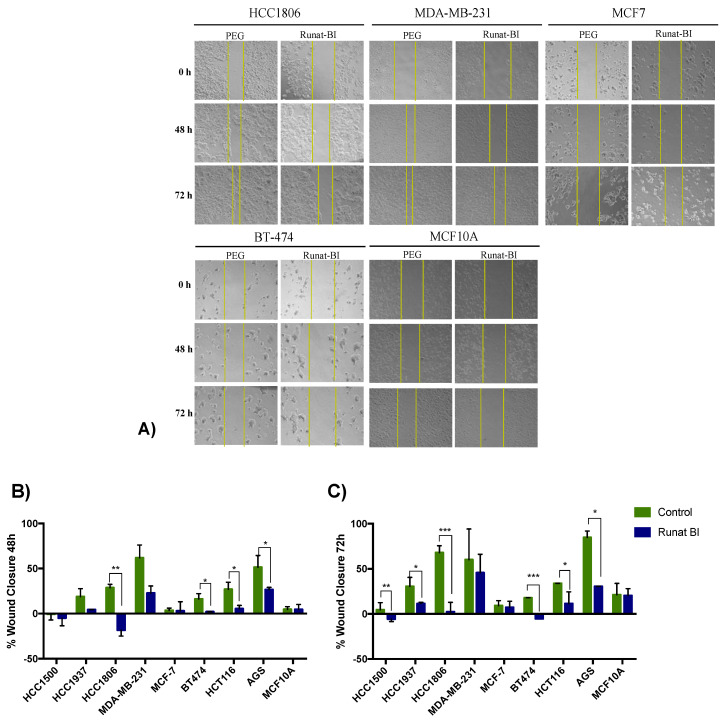
Effect of Runat-BI treatment on cell migration. Cell migration of HCC1806, MDA-MB-231, AGS, HCT116 and MCF10A lines was measured by scratch/wound-healing assay after treatment with Runat-BI (21 µM) or PEG/control for 48 and 72 h. (**A**): Images of cell migration at 0, 48 and 72 h after Runat-BI (21 µM) treatment or PEG (control). Three separate experiments were performed, and the most representative results are presented (5× amplification). Columns express the mean ± SD of the percentage of closure in three independent experiments for 48 h (**B**) and 72 h (**C**). Green bars represent control/PEG and dark blue bars represent Runat-BI-treated cells. * *p* ≤ 0.1, ** *p* ≤ 0.05, *** *p* ≤ 0.01 were considered statistically significant.

All cell lines were treated with 21 µM of Runat-BI. The concentration was established according to the results observed in the viability assays, which showed a block in viability reduction for doses higher than 21 µM. A scratch was performed at 0 h and the percentage of wound closure was measured using images from 0 to 72 h (Figure 3A and Figure S4). 

Wound healing assays demonstrated that Runat-BI significantly reduced cell migration in the HCC1806, AGS, HCT116 and MDA-MB-231 cell lines. More specifically, the HCC1806 cell line showed a reduction in migration of ~47.1% at 48 h and ~65.7% at 72 h. AGS also exhibited a significant decrease, mainly at 72 h, reaching approximately 54.3%. This cell line maintained its high growth rate and CDT but showed a substantial reduction in cell migration. No significant differences in cell migration were found in HCT116 between 48 h and 72 h, and the wound remained between 21 and 22% from closure in both cases. However, a significant reduction in cell growth rate was observed in the sample treated with Runat-BI compared to PEG. These results agree with those previously observed in MTT assays, where these three lines showed a significant drop in cell viability with Runat-BI treatment. MDA-MB-231 exhibited greater reduction in cell viability at 48 h than at 72 h (approximately 39.1% and 14.5%, respectively). Given the fast growth of this line, the wound was almost closed with both treatments, as observed in Figure 3A. The 21 µM concentration of Runat-BI at 72 h was probably not sufficient for this cell line, as indicated by the MTT results. BT474 showed hardly any reduction in cell migration, although it experienced prominent cell death. MCF-7, HCC1937 and HCC1500 showed both less viability reduction and low impact on cell migration when treated with Runat-BI (Figure S4). In MCF10A no differences in cell migration were found between Runat-BI treatment and control (Figure 3A).

The results obtained in cell migration assay support those obtained in viability assays, with the HCT116, AGS and HCC1806 cell lines clearly showing the most important cytotoxic effect from Runat-BI. The selective action against tumor lines compared with the non-tumor cell line (MCF10A) is again demonstrated by the results obtained in the wound healing assay, which showed no migration differences between treatment and control condition (Figure 3B,C). 

Figure S5 shows the comparison of cell migration at 48 h and 72 h in the five lines showing statistical significance in either PEG or Runat-BI, or in both. Generally, the fastest growing lines were more successful in closing the scratch in the control samples at 48 and/or 72 h. However, none of the tumoral cell samples treated with Runat-BI were able to completely close the scratch, demonstrating a clear reduction in cell invasiveness, and in some cell lines, such as BT474, HCC1806 and HCT116, pronounced cell death.

### 3.5. Relationship between Runat-BI Effect and Cell Growth Rate

Ru-based compounds target various DNA conformations and affect DNA processing enzymes. Runat-BI may intercalate in DNA, with the ability to cause DNA condensation and cleavage, inducing cell cycle arrest and/or apoptosis [20]. Cancer cells present higher division rates and are consequently more exposed to the effect of anticancer drugs that act on DNA. Due to the faster growth rates of cancer cell lines, we sought to analyze the possible relationship between CDT and Runat-BI effectiveness. 

**Figure 4:**
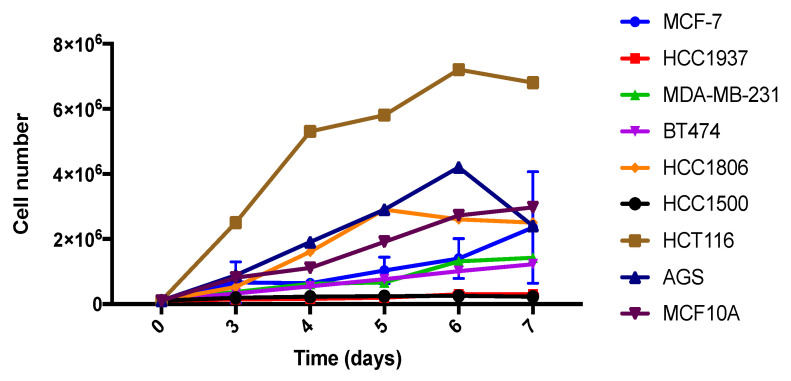
Cell growth over 7 days in cell lines treated with Runat-BI. Cell growth was measured with the Neubauer chamber using “Automated Cell Counter TC10^TM^”. Dots indicate the mean of two independent experiments.

HCT116, HCC1806 and AGS cell lines showed lower CDT rates (Figure 4). Interestingly, these three cell lines displayed higher viability and migration reduction when they were exposed to Runat-BI, indicating that the compound has higher effectiveness against cell lines with higher growth rates. An intermedia effect of Runat-BI was observed for cell lines with intermedia growth rates, such as BT474 and MDA-MB-231. Consequently, cell lines with slow growth rates (higher number of CDT days), such as HCC1500 and HCC1937, exhibited low or absent treatment effect with Runat-BI. However, the relationship is not completely clear since MCF-7 is relatively fast dividing yet Runat-BI displays no significant effect on this cell line. A similar situation is observed with MCF10A, which despite undergoing fast division, is resistant to Runat-BI. These findings partially support that Runat-BI acts against cells with higher growth rates due to their mechanism of biological action. However, we hypothesize the existence of some additional mechanism(s) leading to the selectivity displayed in some cancer cell lines, and which would explain the lack of effectiveness against luminal A subtype MCF-7 and non-tumoral cell line MCF10A.

### 3.6. Apoptosis Induced by Runat-BI Treatment

**Figure 5:**
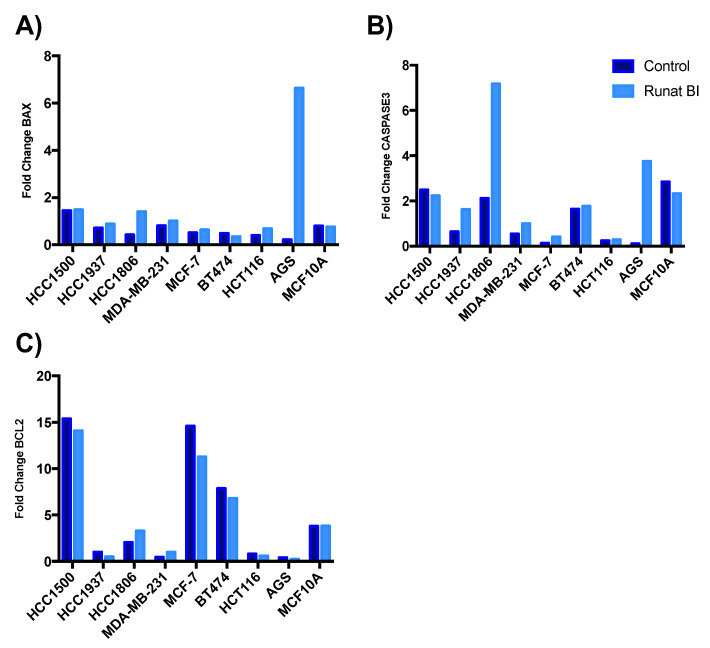
Expression of proapoptotic genes *BAX* (**A**), *CASPASE-3* (**B**) and the antiapoptotic gene *BCL-2* (**C**) in cancer cell lines after Runat-BI treatment. Fold-change (FC) results were obtained by quantitative RT-PCR for the BC lines HCC1500, HCC1937, HCC1806, MDA-MB-231, MCF-7, BT474; colon cancer line HCT116, gastric cancer line AGS and non-tumor line MCF10A. Dark blue represents control/PEG-treated samples and light blue represents Runat-BI-treated samples.

Ruthenium-based complexes may induce apoptosis in cancer cells, acting as potential cancer treatments [21]. Given that most ruthenium-based agents are multitarget, we investigated whether Runat-BI had a different target apart from binding to nuclear DNA and subsequently interference with the transcription. We studied the apoptotic induction of Runat-BI in the nine cell lines, analyzing the expression of the proapoptotic genes *BAX* and *CASPASE-3* and the antiapoptotic gene *BCL-2*. Our results indicated an increase in *BAX* and *CASPASE-3* in most cell lines treated with Runat-BI compared with control samples (Figure 5A,B), although this did not reach statistical significance as analyzed by *t*-test (*p*-value 0.2 for *BAX* and 0.19 for *CASPASE-3*). However, the non-tumoral cell line MCF-10A did not present gene expression deregulation between the two conditions. Slight downregulation of *BCL-2* was observed in some cell lines treated with Runat-BI, but these results were not significant (*p*-value 0.84) [Figure 5C]. Therefore, Runat-BI slightly increases expression of the proapoptotic genes *BAX* and *CASPASE-3* but seemingly fails to affect expression of the antiapoptotic gene *BCL-2*.

## 4. Discussion

In the search for more effective metal-based antitumor agents with fewer adverse effects, those based on ruthenium have attracted particular research interest [22,23,24,25]. Ruthenium complexes have emerged as potential pro-oxidant and multitarget chemotherapeutics to replace platinum-based drugs [26]. We have synthesized a novel compound: Runat-BI, a ruthenium(III) and 2,2’-biimidazole racemic compound, a mixture of two isomers (1:1), of which one (isomer 1) has been isolated. Among the advantages of complexes based on ruthenium(III) with 2,2’-biimidazole are the interactions between nucleobase pairs, which further facilitate the mechanism of action with cancer cell DNA. Furthermore, the ruthenium(III) structure allows them to be more robust and inert when reacting in redox processes or ligand substitution reactions, thus increasing stability and extending the shelf-life of the compound in the physiological environment [27,28]. Ruthenium compounds can carry a high number of potential accessory molecules and show the possibility to exist in biological fluids in almost all the most important oxidation states, from II to IV [29,30].

The results of our experiments with Runat-BI showed a considerable reduction of viability and migration in three cell lines, from three different cancer types: HCT116, from colon cancer, AGS from gastric cancer and HCC1806 from BC. These three cell lines had the lowest CDT rates, proving that Runat-BI is especially active against highly aggressive tumors. Moreover, the reduction in cell migration might prevent tumor expansion to other tissues and thus inhibit metastasis. The IC_50_ results obtained with Runat-BI showed to be similar to those of cisplatin; the cell lines that displayed lower values for cisplatin also display them for Runat-BI, which seems to indicate that both compounds share some mechanism of action.

The compound did not show great effectiveness against HCC1500, HCC1937 and MCF-7, all belonging to BC. Furthermore, BC cell lines belonging to the TNBC subtype (HCC1806 and MDA-MB-231) and luminal B (BT474) exhibited a greater response to Runat-BI treatment than those belonging to the luminal A subtype, such as MCF-7, which showed less remarkable results in both viability and migration assays. Despite the lack of specificity of Runat-BI for a particular cancer type, a selective effect has been observed between non-tumorigenic cell lines such as MCF10A and tumorigenic cell lines. The MCF10A cell line was not affected by the agent, showing no reduction in viability or migration. Similar results in the selectivity of action in non-tumor cell lines have already been demonstrated with other ruthenium agents [16,22,25,31]. Popolin et al. studied four ruthenium-based antitumor complexes with different ligands, among which [Ru(CH_3_CO_2_)(dppb)(bipy)]PF_6_ [where dppb = 1,4-bis(diphenylphosphino)butane and bipy = 2,2’-bipyridine] showed the best results and was selected to be studied in three BC cell lines: MDA-MB-231, MCF-7 and MCF-10A. This complex was more efficient in inhibiting proliferation, adhesion, migration and invasion in MDA-MB-231 cells than non-tumor cells (MCF10A). Nevertheless, the Ru(III) complex showed inconsistent results in MCF-7 [29]. Similarly, Naves et al. reported a selective effect of a ruthenium-biphosphine complex containing gallic acid as a ligand, with selective cytotoxicity against the TNBC cell line (MDA-MB-231) over non-tumor cells (MCF10A), inhibiting its migration and invasion and inducing apoptosis [16,25]. Our in vitro studies support the use of Runat-BI as a potent drug candidate for highly aggressive and proliferative cancers.

Despite the anticancer properties found in Runat-BI, no significant results were obtained for isolated isomer 1 from this complex, which is therefore not solely responsible for its antitumor action. Work is currently underway on the isolation and purification of the second isomer to verify whether Runat-BI agent requires both isomers to exert its action or if only one is responsible for the activity.

There seems to be a relationship between Runat-BI effectiveness and the growth rate of cell lines, since the fastest growing ones generally show the greatest response, probably due to the binding to DNA, which has been considered the main target of action of the agent. However, the relationship is still unclear, as Ru-based drugs seem to exert their anticancer effects through a multitarget mechanism [26], which makes these agents less prone to drug resistance [32,33]. Several studies have revealed that DNA is not necessarily the primary target of ruthenium compounds but rather that these compounds have shown a higher affinity for DNA-related proteins [12,17,32]. In our study, MCF-7 and MCF10A were not affected by Runat-BI despite their rapid growth rates, warranting exploration in future studies of other mechanism(s) of action potentially at play.

We further investigated the expression of proapoptotic genes *BAX* and *CASPASE-3* and the antiapoptotic gene *BCL-2.* Our results showed that Runat-BI upregulated the expression of proapoptotic genes *BAX* and *CASPASE-3* (although no statistically significant differences were obtained) but exerted no effect the antiapoptotic gene expression of *BCL-2*. This is in agreement with results obtained in previous studies on BC cell lines [16,29]. However, the cell lines with the highest rise in antiapoptotic gene expression were not the ones with the most compromised viability, thus potentially ruling this out as the main factor underlying this increased gene expression and indicating a need for further studies for a more precise understanding of this possible pathway of action.

Runat-BI has shown very positive in vitro results, better even than other earlier reported ruthenium systems [34]. Nevertheless, in vivo trials are also necessary to confirm its selective action and possible indications. Another interesting focus would be to study Runat-BI in combination with other commercialized therapeutic agents to evaluate the possible benefits of several targets of action and their joint effect. The combination of previously reported ruthenium compounds with standard anticancer agents that target DNA has shown to be a reasonable combination strategy, particularly in TNBC [35]. 

## 5. Conclusions

Ruthenium-based antitumor agents such as Runat-BI are an interesting alternative to current chemotherapy agents, overcoming many disadvantages of the latter due to their specific activity against different cancers, more favorable toxicity profile and multitargeted mechanism of action. Altogether, our novel complex Runat-BI significantly reduced viability and migration in three cancer cell lines taken from colon, gastric and breast cancer, all of which displayed fast proliferation rates. This supports the effectiveness of the complex in highly proliferative and aggressive cancers that have limited available therapeutic options. Its selective action between tumor and non-tumor lines as well as its potential multitargeted effect on DNA and upregulation of proapoptotic gene expression make it a potential drug candidate for cancer treatment. The effectiveness of the racemic mixture Runat-BI is not due to the isolated isomer 1, and hence further studies should be addressed at evaluating the role of isomer 2 to more precisely elucidate the anticancer potential of Runat-BI and its mechanism of action.

## 6. Patents

Runat-BI has the international patent certificate PCT/ES2022/070415, Universitat de València and Fundación INCLIVA (2021): Ruthenium-biimidazole compound (RUNAT-BI) and its therapeutic use. It has been registered in *Oficina Española de Patentes y Marcas* (OEPM) with No. P202130624. https://www.uv.es/oferta-cientifico-tecnologica/es/resultados/salud/salud-lo-largo-todo-ciclo-vital-1286222426775/OCTResultats.html?id=1286236582025 (accessed on 15 November 2022).

## Data Availability

The data provided in this study are available from the corresponding author on reasonable request.

## Supplementary Materials

The following supporting information can be downloaded at: https://www.mdpi.com/article/10.3390/cancers15010069/s1. Figure S1: Electrospray ionization mass spectrum (ESI-MS) for Runat-BI showing the isotopic distribution for the [RuCl_2_(H_2_biim)_2_]^+^ cation. Figure S2: FT-IR spectra for 2,2’-biimidazole (H_2_biim) and Runat-BI. Table S1: Crystal data and structure refinement for Runat-BI. Table S2: Selected bond lengths (Å) and angles (°) for Runat-BI. Table S3: Cell line characteristics and culture conditions. BC: breast cancer; EGFR: epidermal growth factor receptor; EGP2: epithelial glycoprotein 2; ER: estrogen receptor; FBS: fetal bovine serum; HER2: hormonal estrogen receptor 2; IDC: invasive ductal carcinoma; IGFBP: insulin growth factor binding protein; L-glu: L-glutamine; PR: progesterone receptor; RPMI: RPMI 1640 medium; TGF-β/α: transforming growth factor β/α. *MCF10A is non-tumorigenic human mammary epithelial cells. Table S4: IC_50_ in μM of the nine cancer cell lines studied after 48 h and 72 h of Runat-BI treatment and of Cisplatin. The R square indicates the goodness of fit. IC_50_: half maximal inhibitory concentration. Figure S3: Percentage of viability for AGS and MDA-MB-231 cell lines treated with isomer 1 of Runat-BI at 24, 48 and 72 h. The gastric cancer cell line: AGS and the BC cell line: MDA-MB-231, were treated with isomer 1 of Runat-BI (from 0 to 50 µM) for 24 h (A), 48 h (B) and 72 h (C). Cell proliferation was determined with the MTT assay. Dots indicate the mean of two independent experiments. Figure S4: Effect of Runat-BI treatment on cell migration. Cell migration in HCC1500, HCC1937, MCF-7 and BT-474 cell lines was measured via wound-healing assay after treatment with Runat-BI (21 µM) or control/PEG for 48 and 72 h. Images of cell migration at 0, 48 and 72 h after Runat-BI (21 µM) treatment or PEG (control). Three separate experiments were performed, and the most representative results are presented (5× amplification). Figure S5: Comparison of percentage of wound closure of cell lines treated with Runat-BI (21 µM) or control/PEG for 48 h (green) and 72 h (blue). Columns express the mean ± SD of the percentage of closure in three independent experiments by cell line. * *p* ≤ 0.1, ** *p* ≤ 0.05, *** *p* ≤ 0.01 were considered statistically significant.
