# Supplementary material for: RUNAT-BI: A Ruthenium(III) Complex as a Selective Anti-Tumor Drug Candidate against Highly Aggressive Cancer Cell Lines
Source: Cancers (Basel). 2022 Dec 22;15(1):69. doi: 10.3390/cancers15010069 (PMC9817854; doi:10.3390/cancers15010069)
Supplement: Supplementary file 1 [file cancers-15-00069-s001.zip › cancers-2053223-supplementary.pdf]

Supplementary Materials

RUNAT-BI: a ruthenium(III) complex as a selective antitumoral drug candidate against highly aggressive cancer cell lines

Marta Albanell-Fernández[1,†], Sara S. Oltra[1,†], Marta Orts-Arroyo[2], Maider Ibarrola-Villava[1,3], Fany Carrasco[1,3], Elena Jiménez-Martí[1,3,4], Andrés Cervantes[1,3], Isabel Castro[2], José Martínez-Lillo[2,*], Gloria Ribas[1,3,*]

[1]Biomedical Research Institute INCLIVA, Hospital Clínico Universitario Valencia, University of Valencia, Spain
[2]Instituto de Ciencia Molecular (ICMol)/Departament de Química Inorgànica, University of Valencia, Spain
[3]Center for Biomedical Network Research on Cancer (CIBERONC)
[4]Departament de Bioquímica i Biología Molecular, Facultat de Medicina, University of Valencia, Spain

**Correspondence: gribasdespuig@gmail.com and f.jose.martinez@uv.es; Tel.: +34-9635-44460*

Table of contents page

Figure S1...2

Figure S2...3

Table S1...4

Table S2...4

Table S3...5

Table S4...5

Figure S3...6

Figure S4...7

Figure S5...8

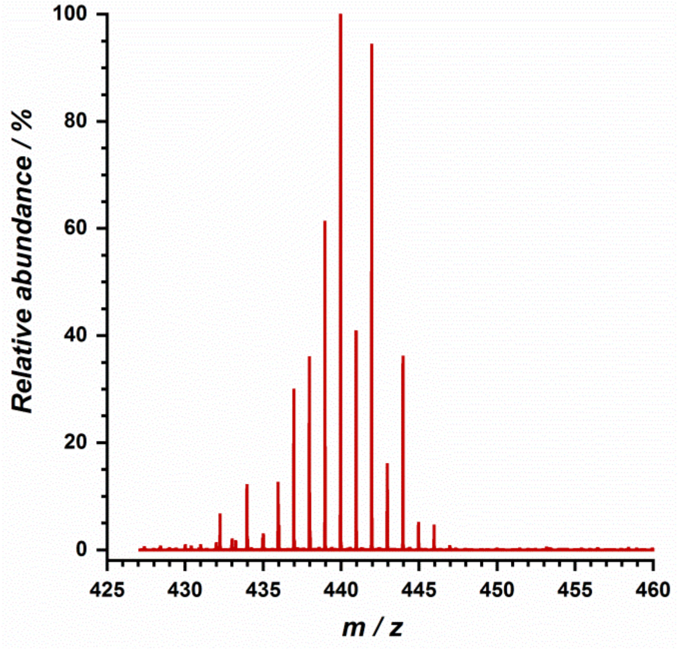

Figure S1. Electrospray ionization mass spectrum (ESI-MS) for Runat-BI showing the isotopic distribution for the $[RuCl_2(H_2biim)_2]^+$ cation with *m/z*: 439.97 (100%).

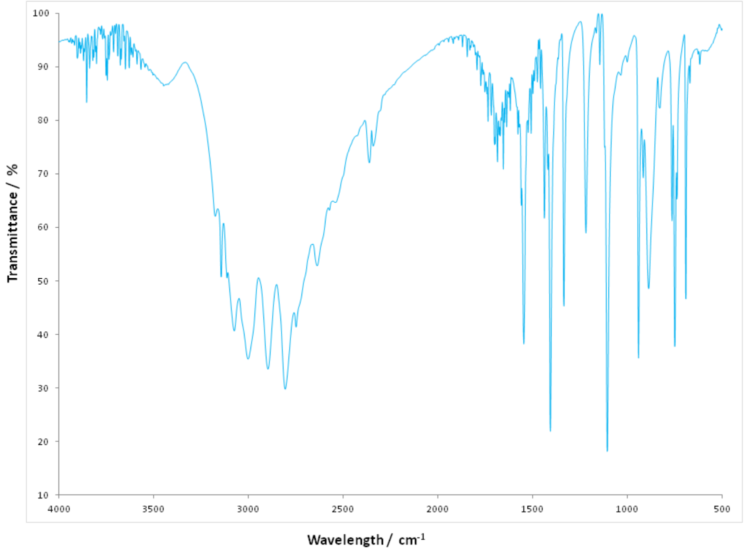

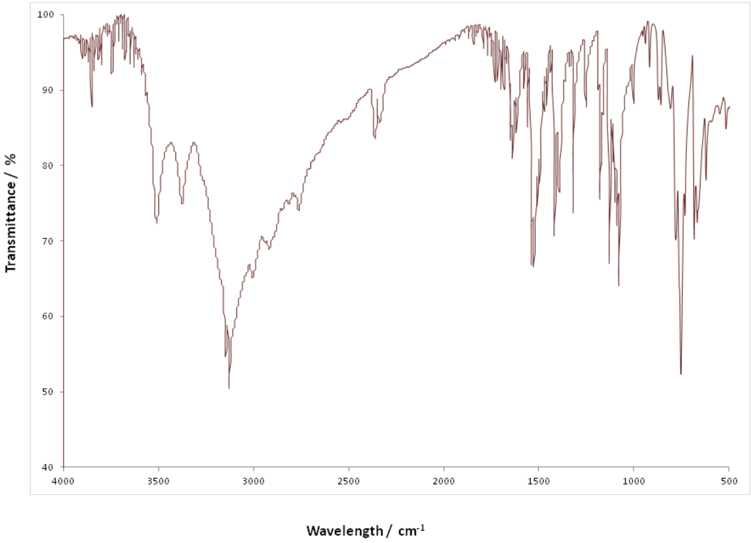

Figure S2. FT-IR spectra for 2,2’-biimidazole (H_2biim, top) and Runat-BI ({*cis*-[$RuCl_2(H_2biim)_2$]Cl}$_2\cdot 4H_2O$, bottom).

Table S1. Crystal data and structure refinement for Runat-BI.

Compound	Runat-BI
Formula	$C_{12}H_{12}N_8O_2Cl_3Ru$
M_r/g mol^{-1}	507.72
Crystal system	monoclinic
Space group	$P2_1$
a/Å	13.457(1)
b/Å	11.317(1)
c/Å	13.749(1)
α/°	90
β/°	115.6(1)
γ/°	90
V / Å^3	1888.14(1)
Z	4
D_c/g cm^{-3}	1.785
μ(Mo-K$_\alpha$)/mm^{-1}	1.279
F(000)	1004
Goodness-of-fit on F^2	1.073
R_1 [$I > 2\sigma(I)$]	0.0423
wR_2 [$I > 2\sigma(I)$]	0.1301

Table S2. Selected bond lengths (Å) and angles (°) for Runat-BI.

Bond lengths	
Ru1-Cl5	2.362
Ru1-Cl6	2.340
Ru1-N1	2.050
Ru1-N2	2.075
Ru1-N5	2.055
Ru1-N6	2.069
**Angles**	
Cl5-Ru1-Cl6	92.97
Cl5-Ru1-N1	90.48
Cl5-Ru1-N2	91.72
Cl5-Ru1-N5	169.60
Cl5-Ru1-N6	92.48
Cl6-Ru1-N1	176.00
Cl6-Ru1-N2	92.75
Cl6-Ru1-N5	92.21
Cl6-Ru1-N6	90.66

Bond lengths	
Ru2-Cl3	2.362
Ru2-Cl4	2.340
Ru2-N9	2.050
Ru2-N10	2.075
Ru2-N14	2.055
Ru2-N14	2.069
**Angles**	
Cl3-Ru2-Cl4	96.19
Cl3-Ru2-N9	85.84
Cl3-Ru2-N10	170.85
Cl3-Ru2-N13	94.13
Cl3-Ru2-N14	118.65
Cl4-Ru2-N9	169.60
Cl4-Ru2-N10	92.96
Cl4-Ru2-N13	88.98
Cl4-Ru2-N14	92.12

Table S3. Cell line characteristics and culture conditions.

Cell line	Cancer type	Cancer Subtype	Receptor expression	Tumor type	Culture medium	Conditions	Supplements
HCC1500	BC	Luminal A	ER,PR	IDC	RPMI	5%CO_2 37 ℃	1% L-glu 10% FBS
HCC1937	BC	Basal	EGP2	IDC	RPMI	5%CO_2 37 ℃	1% L-glu 10% FBS
MDA-MB-231	BC	Basal	EGFR, TGF-β	Carcinoma	RPMI	5%CO_2 37 ℃	1% L-glu 10% FBS
MCF-7	BC	Luminal A	ER, IGFBP	IDC	RPMI	5%CO_2 37 ℃	1% L-glu 10% FBS
BT474	BC	Luminal B	ER, PR, HER2	IDC	DMEM	5%CO_2 37 ℃	1% L-glu 10% FBS
HCC1806	BC	Basal	EGP2	Carcinoma	RPMI	5%CO_2 37 ℃	1% L-glu 10% FBS
AGS	Gastric cancer	-	-	Adenocarcinoma	DMEM	5%CO_2 37 ℃	1% L-glu 10% FBS
HCT116	Colon cancer	-	-	Carcinoma	DMEM	5%CO_2 37 ℃	1% L-glu 10% FBS
MCF10A	*Mammary epithelial	-	-		DMEM/ F-12	5%CO_2 37 ℃	1% L-glu 10% FBS

BC: Breast cancer; EGFR: Epidermal growth factor receptor; EGP2: Epithelial glycoprotein 2; ER: estrogen receptor; FBS: fetal bovine serum; HER2: hormonal estrogen receptor 2; IDC: Invasive ductal carcinoma; IGFBP: Insulin growth factor binding protein; L-glu: L-glutamine; PR: progesterone receptor; RPMI: RPMI 1640 medium; TGF-β / α: transforming growth factor β / α. *MCF10A is non-tumorigenic human mammary epithelial cells.

Table S4. IC_{50} in μM of the nine cancer cell lines studied after 48 h and 72 h of Runat-BI treatment and Cisplatin. The R square indicates the goodness of fit. IC_{50}: half maximal inhibitory concentration.

Cell line	IC_{50} (μM) 48h	R (48h)	IC_{50} (μM) 72h	R (72h)	IC_{50} (μM) Cisplatin
**MCF-7**	269.00	0.008946	49.43	-0.1836	100.36
**HCC1937**	94.23	0.401200	65.81	0.4829	133.50
**MDA-MB-231**	107.40	0.822000	48.89	0.7822	43.15
**BT474**	68.78	0.794400	32.63	0.8595	1140.20
**HCC1806**	24.55	0.970300	27.62	0.8830	9.29
**HCC1500**	76.03	0.127000	192.3	0.3431	97.28
**HCT116**	22.43	0.918100	15.54	0.8630	14.45
**AGS**	43.53	0.746200	29.57	0.6740	13.37

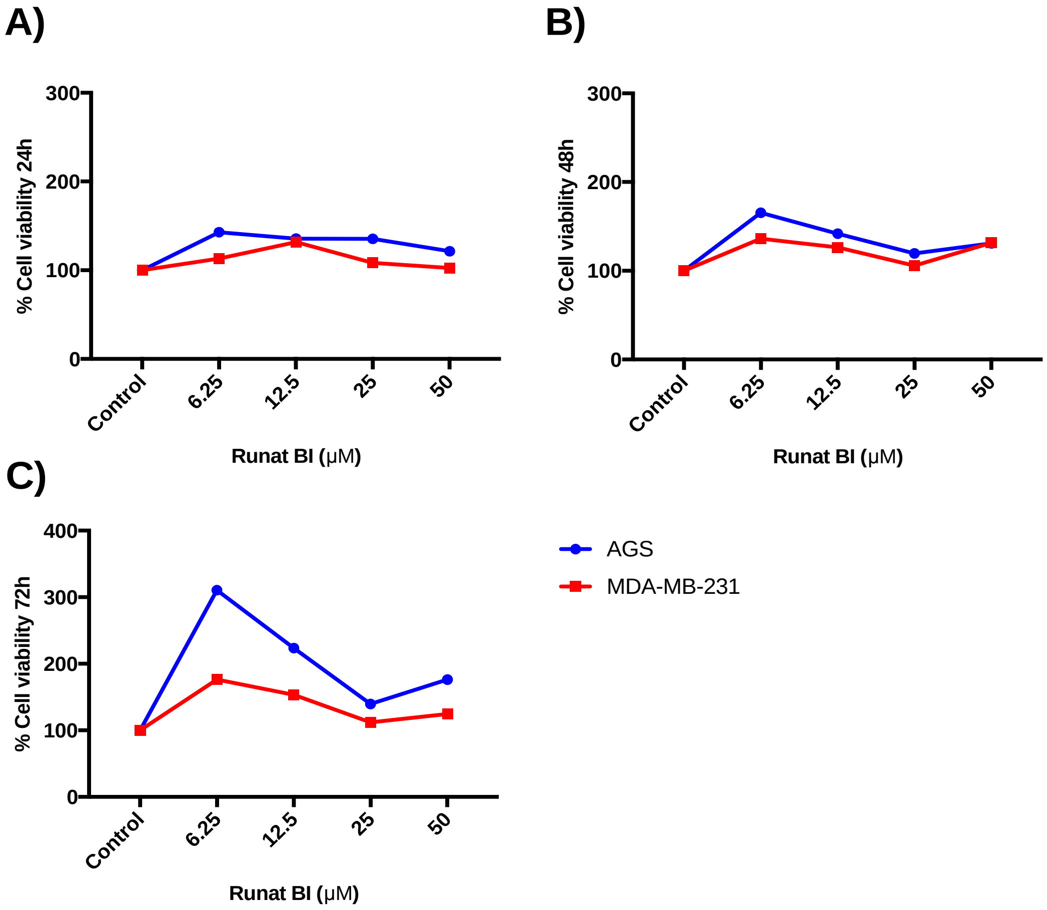

Figure S3. Percentage of viability for AGS and MDA-MB-231 cell lines treated with isomer 1 of Runat-BI at 24, 48 and 72 h. The gastric cancer cell line AGS and the BC cell line MDA-MB-231 were treated with isomer 1 of Runat-BI (from 0 to 50 μM) for 24 h (A); 48 h (B) and 72 h (C). Cell proliferation was determined with the MTT assay. Dots indicate the mean of two independent experiments.

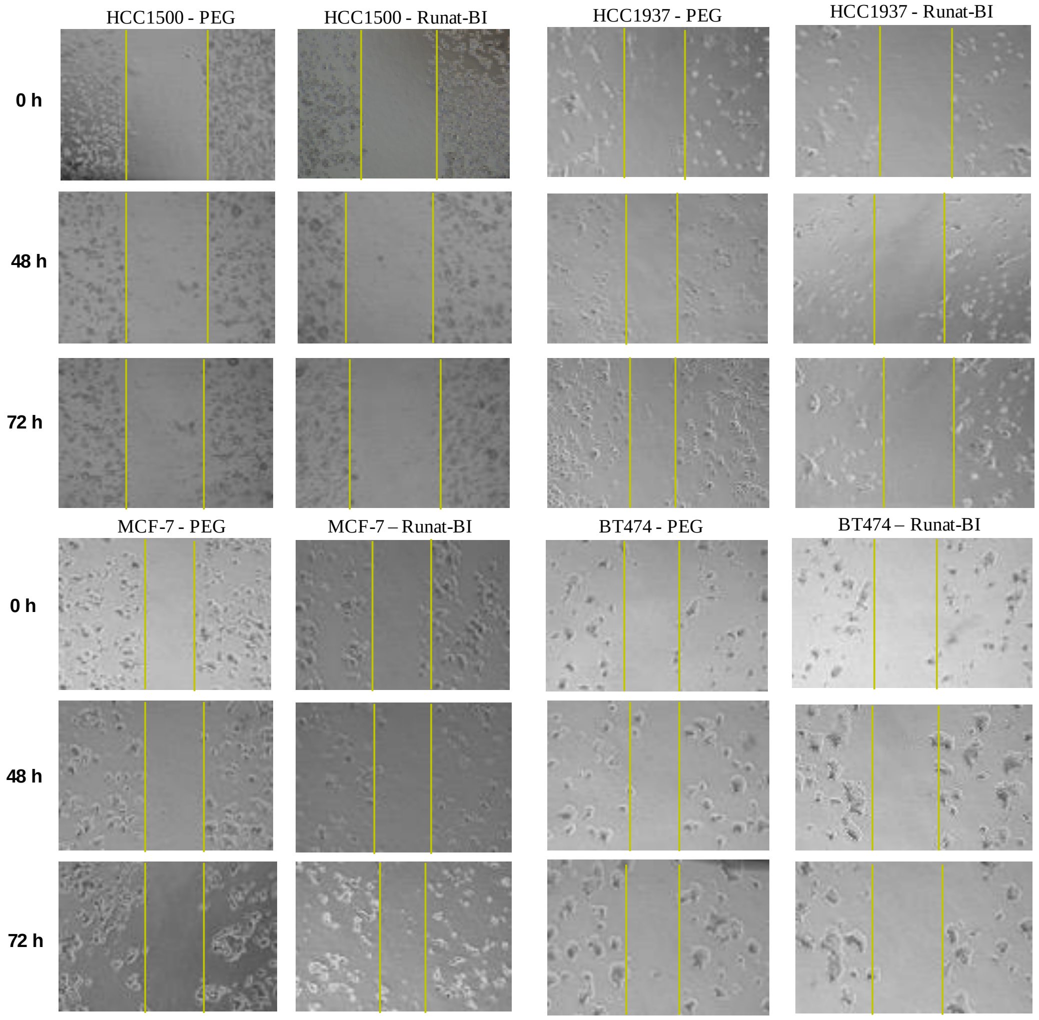

Figure S4. Effect of Runat-BI treatment in cell migration. Cell migration in HCC1500, HCC1937, MCF-7 and BT-474 cell lines was measured with the "wound-healing" assay after treatment with Runat-BI (21 µM) or control/PEG for 48 and 72 h. Images of cell migration at 0, 48 and 72 h after Runat-BI (21 µM) treatment or PEG (control). Three separate experiments were performed and the most representative results are presented (5x amplification).

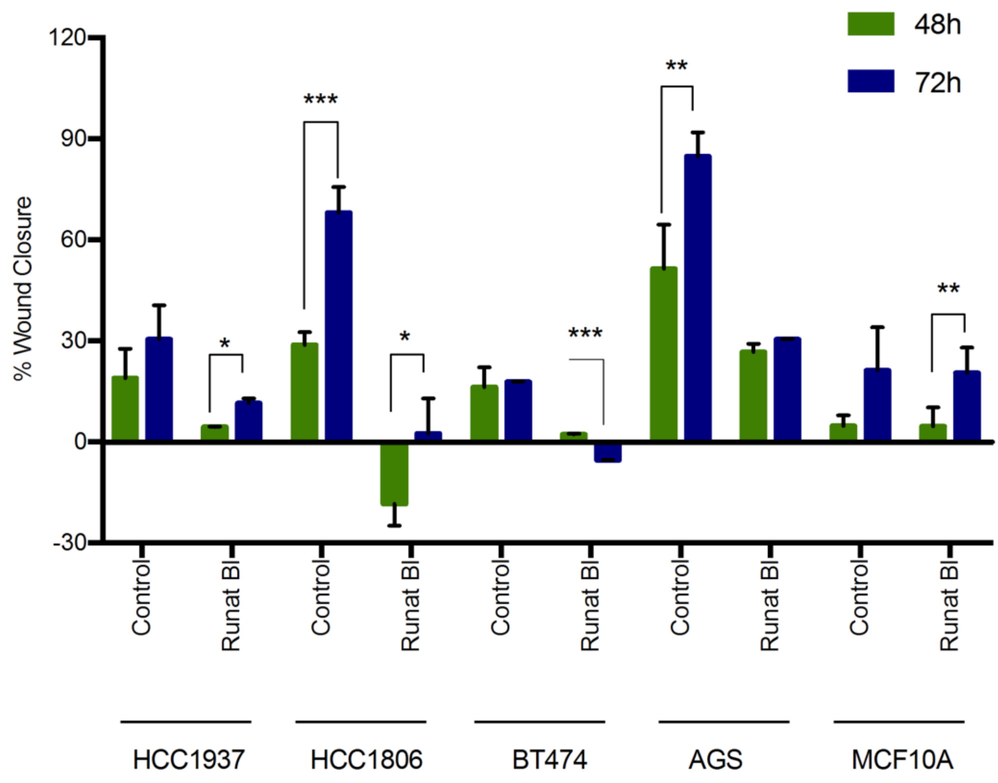

Figure S5. Comparison of percentage of wound closure of cell lines treated with Runat-BI (21 µM) and control/PEG for 48 h (green) and 72 h (blue). Columns express the mean ±SD of the percentage of closure in three independent experiments by cell line. *$P \leq 0.1$, **$P \leq 0.05$, ***$P \leq 0.01$ statistically significant.
